# Radiographic Verification of the Feasibility of Intramedullary Nailing in Tibial Shaft Fractures Distal to Total Knee Arthroplasty

**DOI:** 10.3390/jcm15051801

**Published:** 2026-02-27

**Authors:** Jaewoong Um, Hoon-Sang Sohn, DooSup Kim, HoeJeong Chung, Taesoo Kim

**Affiliations:** Wonju Severance Christian Hospital, 20 Ilsan-ro, Wonju 26426, Gangwon-do, Republic of Korea; wodnd82@gmail.com (J.U.); dskim1974@yonsei.ac.kr (D.K.); hjchung29@yonsei.ac.kr (H.C.); ynent9020@naver.com (T.K.)

**Keywords:** tibial fractures, intramedullary nailing, arthroplasty, replacement, knee, tibial tuberosity, radiographic feasibility

## Abstract

**Background**: Tibial shaft fractures distal to total knee arthroplasty (TKA) are commonly treated with plate fixation, which requires prolonged weight-bearing restriction. Although intramedullary nailing (IMN) has been attempted in selected cases, its feasibility remains controversial. This study aimed to assess the radiographic feasibility of IMN using anatomical parameters on lateral knee radiographs. **Methods**: A total of 271 lateral knee radiographs after TKA (January 2022 to October 2023) were retrospectively reviewed. Nail corridor and superior and inferior tibial tuberosity angles were measured on true lateral views. Tibial component size, keel length, BMI, age, and sex were analyzed for their association with nail corridor dimensions. Calibration was performed using known implant sizes. **Results**: Of the 271 lateral knee radiographs reviewed, 248 patients were included in the final analysis. The mean nail corridor was 9.27 ± 2.41 mm. The average superior and inferior tibial tuberosity angles were 105.88° and 155.79°, respectively. The tibial component size and keel length were not correlated with the nail corridor. In contrast, both superior (β = 0.252, *p* < 0.001) and inferior (β = 0.148, *p* = 0.003) tibial tuberosity angles were significantly associated with the nail corridor. No differences were observed in sex or BMI. **Conclusions**: IMN may be radiographically feasible in selected patients with tibial shaft fractures distal to TKA. The superior and inferior tibial tuberosity angles are anatomical parameters associated with the nail corridor and may serve as reference measures during preoperative radiographic assessment.

## 1. Introduction

With the increasing frequency of Total Knee Arthroplasty (TKA) procedures, the incidence of periprosthetic tibial fractures is expected to rise. Compared with periprosthetic femoral fractures, periprosthetic tibial fractures are less common, with an estimated incidence of approximately 1% [1,2]. In a study of over 17,000 TKA cases, the incidence of tibial shaft fractures occurring distal to a well-fixed implant (classified as Mayo 3A, Felix and Associates’ classification of periprosthetic fractures of the tibia associated with TKA Type III) was reported to be 0.09% [3]. Given that the prevalence of TKA in the United States is expected to exceed 7 million cases by 2030 [4], it can be estimated that approximately 6000 cases of tibial shaft fractures distal to TKA will occur annually.

Non-displaced tibial fractures occurring distal to TKA have traditionally been managed nonoperatively, while surgical intervention, when required, has most commonly been performed using plate osteosynthesis [1,5]. In common practice, however, displaced tibial shaft fractures distal to TKA require weight-bearing restriction for several weeks. Prolonged non-weight bearing may lead to serious complications, including pneumonia, embolism, pressure ulcers, and cognitive decline [6].

While intramedullary nailing (IMN) is considered the standard treatment for mid-shaft tibial fractures and remains a widely accepted option for treating proximal and distal diaphyseal tibial fractures, its use in patients with prior TKA has been debated due to technical challenges [7,8,9,10]. Nevertheless, numerous studies have suggested that IMN offers significant advantages, including faster functional recovery, earlier weight bearing, and reduced soft tissue disruption, making it a viable surgical option [6,11,12,13,14,15].

Despite these potential benefits, most existing studies on IMN for tibial fractures distal to TKA consist primarily of case reports and technical descriptions, and the debate continues regarding its feasibility and safety. Therefore, this study aimed to radiographically verify the feasibility of IMN for diaphyseal tibial fractures occurring distal to TKA through radiographic analysis and measurements.

## 2. Methods

This retrospective study initially reviewed 271 patients who underwent TKA between January 2022 and October 2023. After applying the exclusion criteria, 248 patients were included in the final analysis. Demographic data, including age, sex, and body mass index (BMI), were collected. Additionally, radiographic parameters, such as tibial baseplate size, keel length, nail corridor, superior tibial tuberosity angle, and inferior tibial tuberosity angle, were measured.

**Figure 1:**
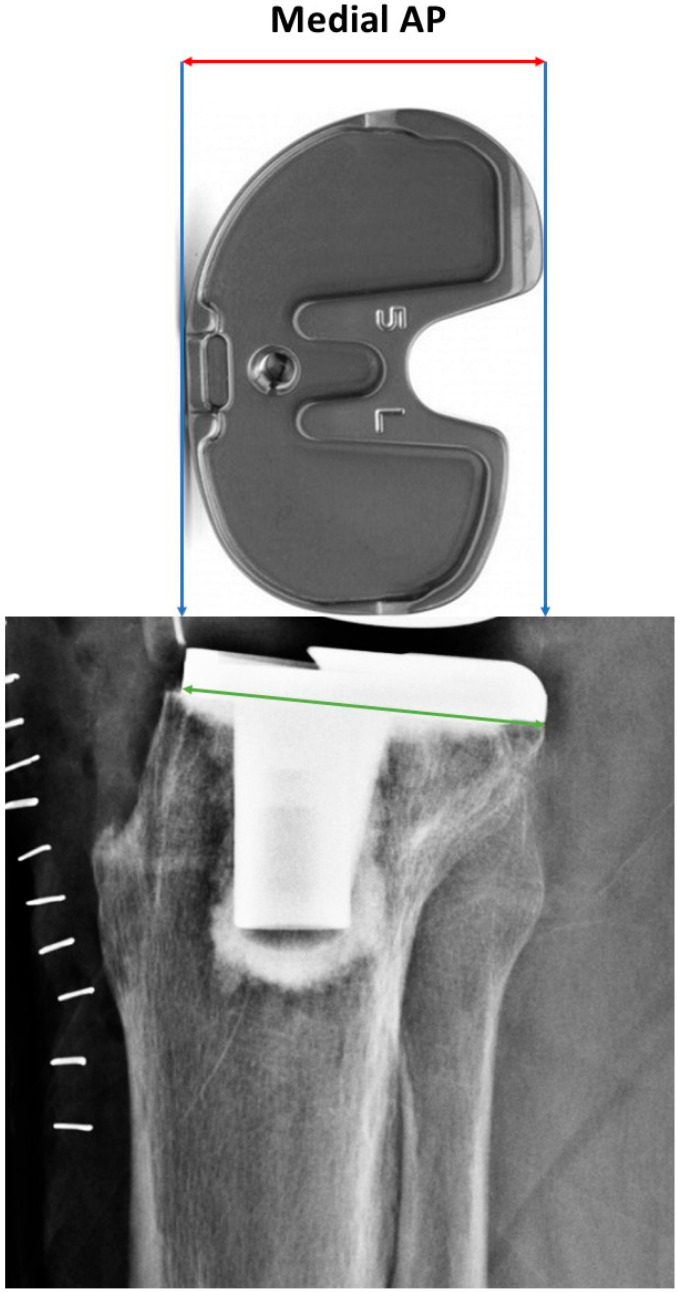
Measurement of medial anteroposterior (AP) width of the tibial component. (**top**) Photograph of the actual tibial implant showing the medial AP length used for calibration. (**bottom**) Corresponding lateral radiograph demonstrating the medial AP line (green) used to calibrate radiographic measurements. The blue lines and red arrows indicate the medial AP dimension of the tibial component used for calibration.

Since actual radiographic measurements may not fully correspond to real anatomical dimensions, a calibration process was performed using the anteroposterior length of the tibial baseplate and the keel length from the real implanted prosthesis to enhance accuracy (Figure 1).

For radiographic analysis, we selected one true lateral knee radiograph in which the femoral component of TKA was perfectly superimposed.

**Figure 2:**
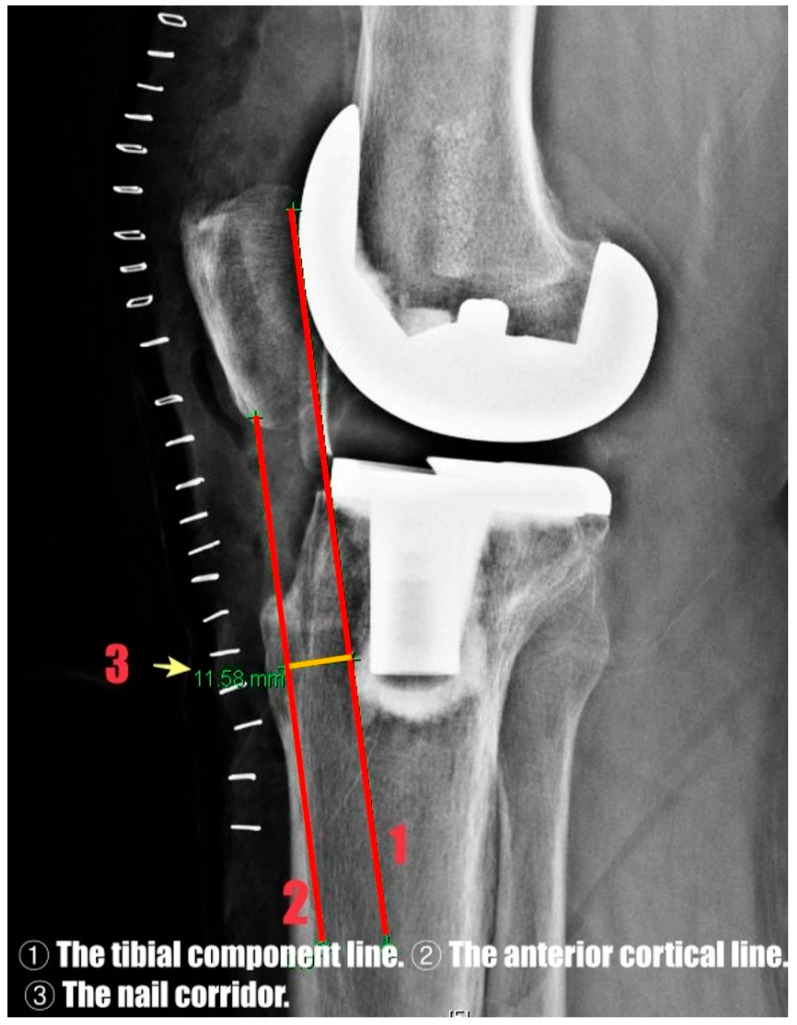
Radiographic definition of the nail corridor on lateral knee radiograph. The red lines represent the tibial component line (1) and the anterior cortical line (2), and the orange segment between them indicates the nail corridor (3).

The nail corridor was defined as the shortest perpendicular distance between the anterior edge of the tibial component (including the cement interface) and the anterior cortical line on the lateral radiograph, representing the available anteroposterior space for intramedullary nail insertion (Figure 2).

**Figure 3:**
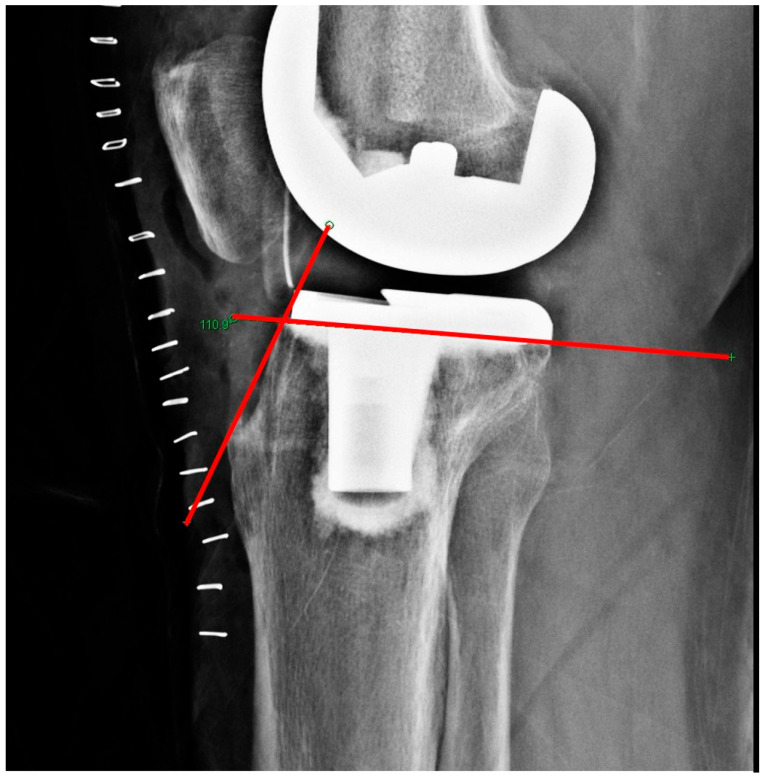
Radiographic measurement of the superior tibial tuberosity angle. The angle is defined by the intersection of the red lines.

**Figure 4:**
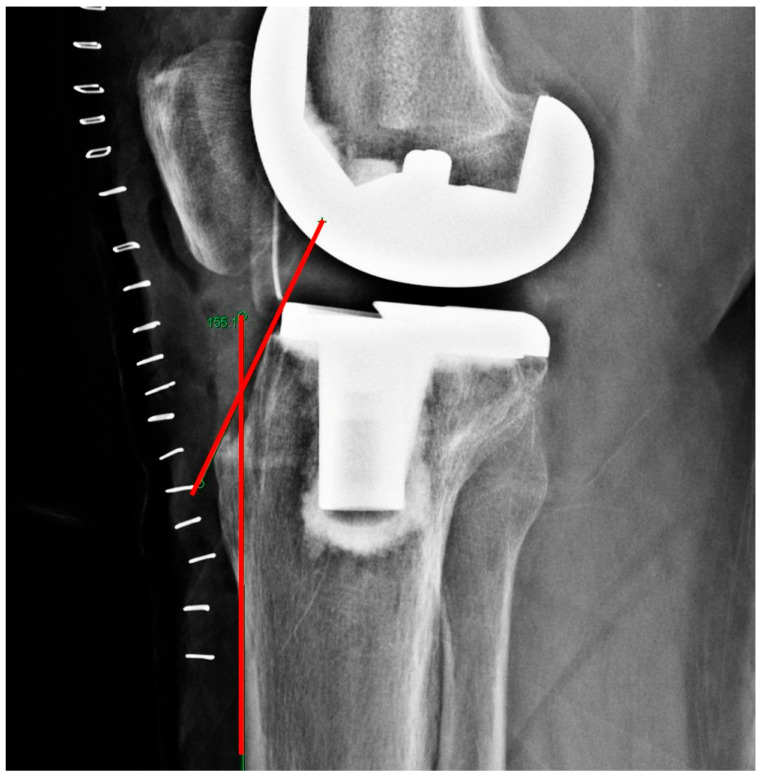
Radiographic measurement of the inferior tibial tuberosity angle. The angle is defined by the intersection of the red lines.

The superior tibial tuberosity angle was defined as the angle between the tibial component and the slope of the tibial tuberosity on the lateral radiograph (Figure 3). The inferior tibial tuberosity angle was defined as the angle between the anterior cortical line of the tibial shaft and the anterior cortical contour of the proximal tibial metaphysis, extending from the anterior margin of the tibial plateau to the level of the tibial tuberosity, on the lateral radiograph (Figure 4).

Patients in whom the anterior border of the medullary canal was inconspicuous (*n* = 4) and those with long-stem implants (*n* = 19) were excluded from the analysis. Radiographic measurements were independently reviewed by the authors. Any disagreement on the values of the parameters was resolved by reaching a consensus. The influence of demographic factors and radiographic parameters on the nail corridor was also evaluated.

### Statistical Analysis

Statistical analyses were performed to identify the factors associated with the nail corridor. The univariate analyses were performed using *t*-test, Pearson correlation, and ANOVA. Variables with *p* < 0.05 were entered into a stepwise multiple regression model.

Multiple linear regression analysis was conducted to evaluate the independent effects of the multiple variables on the nail corridor. A stepwise approach was used to construct a full model incorporating tibial size (as a categorical variable), superior tibial tuberosity angle, and inferior tibial tuberosity angle.

All analyses were performed using IBM SPSS Statistics (version 25.0; IBM Corp., Armonk, NY, USA) and R (version 4.3.1; R Foundation for Statistical Computing, Vienna, Austria) via RStudio (version 2023.09.1). Statistical significance was set at *p* < 0.05.

## 3. Results

A total of 248 patients were included in the analysis, with a mean age of 74.11 ± 6.97 years. The cohort comprised 188 females (75.8%) and 60 males (24.2%). The mean BMI of the cohort was 22.00 ± 4.33 kg/m^2^, indicating that the majority of patients were within the normal weight range.

Among them, 228 patients (91.9%) received the Persona implant, and 20 (8.1%) received the GKS implant. The tibial size distribution varied according to the implant type. The most frequent sizes in the Persona group were C (40%), B (25%), and E (20%), whereas in the GKS group, the predominant sizes were D (39%), C (22.8%), and E (15.4%). The Keel length distribution differed by implant type. The mean superior and inferior tibial tuberosity angles were 105.9 ± 5.9° and 155.8 ± 5.7°, respectively. The mean nail corridor was 9.27 ± 2.41 mm (Table 1).

A sex-based comparison using an independent samples *t*-test revealed that males had a higher mean nail corridor (9.85 mm) than females (9.09 mm), although the difference was not statistically significant (*p* = 0.072, 95% CI: −1.58 to 0.07). The Pearson correlation coefficient showed no significant association between age and nail corridor (r = 0.038, *p* = 0.552), indicating a negligible relationship.

Additionally, the linear regression analysis confirmed that age was not a meaningful associated parameter of the nail corridor (β = 0.013, *p* = 0.552, R^2^ = 0.0014). The BMI also showed no statistically significant association with the nail corridor in either group. In the Persona group, the association was not significant (β = −0.061, *p* = 0.681), and in the GKS group, BMI showed no significant difference (β = −0.054, *p* = 0.686).

In the Persona implant group, tibial size was significantly associated with the nail corridor (*p* = 0.0037, one-way ANOVA). Post hoc analysis using Tukey’s test revealed that tibial size G had a significantly greater nail corridor than sizes C (*p* = 0.0016), D (*p* = 0.0347), and E (*p* = 0.0042).

However, Keel length did not show a statistically significant association with the nail corridor in either implant group. In the Persona group, the relationship was not significant (β = 0.015, *p* = 0.893). Similarly, in the GKS group, no significant association was observed (β = 0.165, *p* = 0.833).

Correlation analysis showed that the superior tibial tuberosity angle was moderately positively correlated with the nail corridor (r = 0.313, *p* < 0.001), whereas the inferior tibial tuberosity angle had a weak negative correlation (r = −0.181, *p* = 0.004).

Simple linear regression revealed a significant positive association between the superior tibial tuberosity angle and nail corridor (β = 0.128, *p* < 0.001, R^2^ = 0.098), whereas the inferior angle was negatively associated (β = −0.076, *p* = 0.004, R^2^ = 0.033).

In the multivariate regression model, including both the superior and inferior tibial tuberosity angles, both parameters remained statistically significant. The superior angle demonstrated a stronger association (β = 0.252, *p* < 0.001), whereas the inferior angle was also significant (β = 0.148, *p* = 0.003). The stepwise model explained 12.2% of the variance (Adjusted R^2^ = 0.122), whereas tibial size lost statistical significance after adjustment. The full model was statistically significant (F (8, 218) = 7.38, *p* < 0.001), and explained 18.4% of the variance (Adjusted R^2^ = 0.184). Scatterplots illustrating the relationships between the superior and inferior tibial tuberosity angles and the nail corridor are provided in Supplementary Figures S1 and S2.

The residuals of the full model were evaluated using a Q–Q plot. The plot exhibited approximate linearity with minor deviations in the tails, indicating that the normality assumption was reasonably satisfied. Assessment of model residuals is shown in Supplementary Figure S3.

Given the adequate sample size (*n* = 248), no further transformations were required. Although both angular parameters showed statistically significant associations with the nail corridor, the overall explanatory power of the multivariate model remained modest, indicating that additional anatomical and implant-related factors may also contribute.

## 4. Discussion

This study evaluated the radiographic feasibility of intramedullary nailing (IMN) for tibial shaft fractures distal to total knee arthroplasty (TKA) using standardized lateral knee radiographs. The mean nail corridor was 9.27 ± 2.41 mm, suggesting that IMN may be radiographically feasible in selected patients rather than universally applicable. In addition, both the superior and inferior tibial tuberosity angles demonstrated statistically significant associations with the nail corridor.

In the univariate analysis, the superior tibial tuberosity angle showed a moderate positive correlation with the nail corridor, whereas the inferior tibial tuberosity angle demonstrated a weak negative correlation. Interestingly, in the multivariate regression model, the inferior tibial tuberosity angle exhibited a positive association after adjustment for the superior angle. This finding suggests a potential interaction or confounding relationship between angular parameters and highlights the importance of multivariate analysis when interpreting the anatomical determinants of radiographic nail feasibility. Nevertheless, the overall explanatory power of the multivariate model was modest, indicating that the identified angular parameters account for only a portion of the variability in nail corridor dimensions and that additional anatomical or implant-related factors are likely involved.

Previous studies have described important technical considerations for IMN after TKA, including the position of the tibial component, the extent of the cement mantle, and the presence of an anterior cortical constraint [12,13,14,15]. Radiographic parameters such as the anterior cortex–implant distance (ACID) described by Stevens et al. and the keel–tuberosity distance reported by Shaath et al. and Devendra et al. share conceptual similarities with the nail corridor assessed in the present study [12,13,15]. However, most prior investigations were limited to technical notes, case series, or descriptive radiographic assessments and did not statistically evaluate which specific anatomical features influence the available nail corridor.

**Figure 5:**
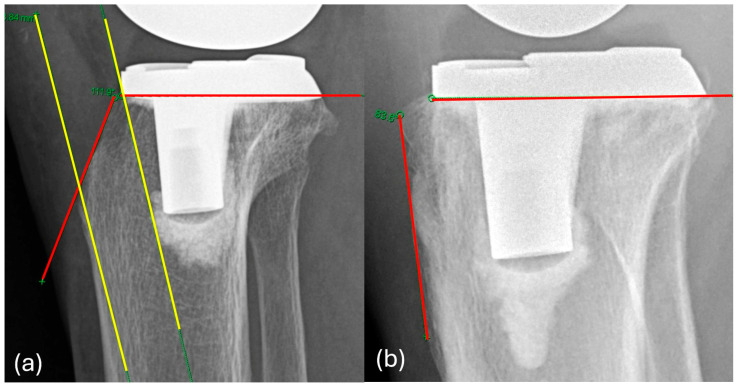
Representative lateral knee radiographs demonstrating the range of superior tibial tuberosity angles encountered in clinical practice. These images are presented for illustrative purposes only and are not intended for direct radiographic comparison. The superior tibial tuberosity angle is defined by the red lines, and the nail corridor is illustrated by the yellow segment. (**a**) A large superior tibial tuberosity angle with a wide nail corridor. (**b**) A small superior tibial tuberosity angle with a markedly narrow corridor.

To our knowledge, this study is the first to statistically identify both the superior and inferior tibial tuberosity angles as anatomical parameters associated with the nail corridor. The superior tibial tuberosity angle may be particularly relevant in the context of the suprapatellar approach, which requires controlled navigation of the nail through the anterior tibial metaphysis. A larger superior angle may provide a more favorable insertion trajectory and reduce the likelihood of contact with the tibial component. In contrast to the superior angle, the inferior tibial tuberosity angle reflects the curvature of the proximal anterior metaphyseal contour and may influence the anterior constraint encountered along the nail trajectory. Although the inferior tibial tuberosity angle demonstrated a weaker individual association, its significance in the multivariate model suggests that it may contribute to compensating for anterior cortical constraints when considered alongside other angular parameters. (Figure 5).

**Figure 6:**
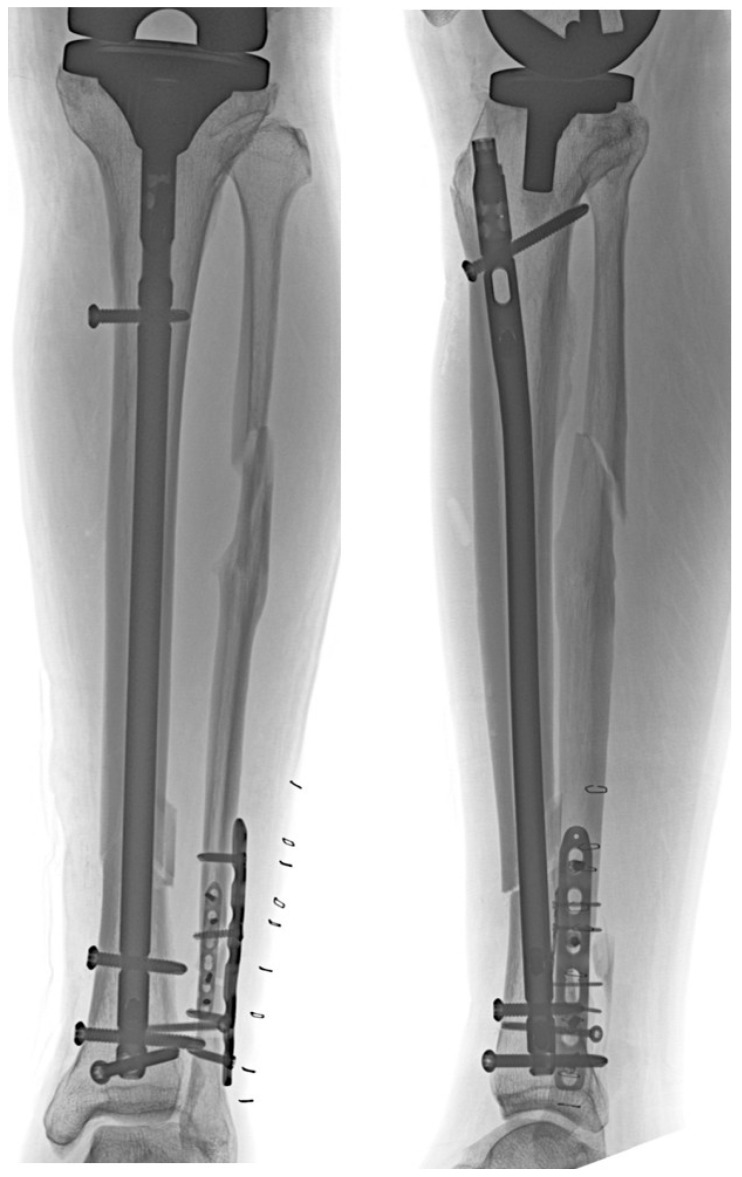
Postoperative anteroposterior and lateral radiographs of an illustrative clinical case in which intramedullary nailing (IMN) was performed for a tibial shaft fracture distal to a total knee arthroplasty (TKA).

**Figure 7:**
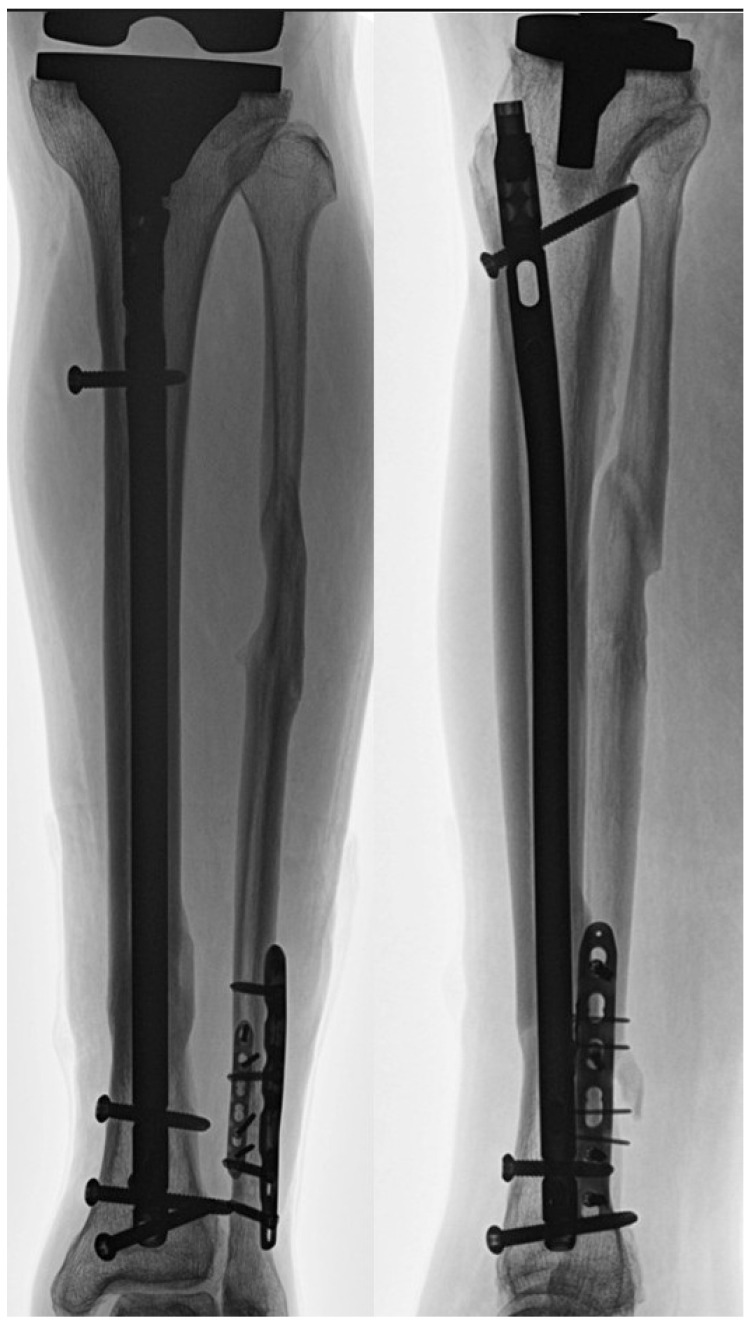
Follow-up radiograph obtained 15 months postoperatively demonstrating fracture union in the illustrative case shown in Figure 5.

Despite these findings, the clinical implications of this study should be interpreted with caution. The observed associations largely reflect expected geometric relationships, and radiographic feasibility does not necessarily equate to intraoperative technical feasibility or clinical success. Accordingly, the superior and inferior tibial tuberosity angles should be regarded as reference parameters rather than definitive predictors of IMN feasibility. Their primary value lies in assisting preoperative radiographic assessment and surgical planning, particularly when IMN is being considered as an alternative to plate fixation in patients with prior TKA. An illustrative clinical case in which IMN was performed and subsequent fracture union was achieved is shown (Figure 6 and Figure 7). Therefore, these findings should be interpreted as radiographic associations rather than direct indicators of intraoperative feasibility. Rather than predicting clinical outcomes, the present findings provide a radiographic framework to identify patients in whom an anterior nail trajectory may be technically achievable without violating the tibial component or anterior cortex.

Several important limitations should be acknowledged. First, this study was based solely on two-dimensional lateral radiographs, which may not fully represent the three-dimensional anatomical space encountered during actual intramedullary nail insertion. Second, no intraoperative findings or clinical outcomes were available to validate whether the measured nail corridor translated into successful nail insertion or improved postoperative outcomes. Third, although statistically significant, the explanatory power of the multivariate model was modest, indicating that the identified angular parameters explain only a limited proportion of the variability in nail corridor dimensions. Additionally, as the study population consisted predominantly of patients from a single ethnic background, anatomical variations related to ethnicity may limit the generalizability of the present findings to other populations. Fourth, the study population predominantly consisted of two implant designs, introducing implant-type selection bias and limiting generalizability to other tibial component geometries.

Although the present study relied on standardized two-dimensional lateral radiographs to ensure reproducibility and broad clinical applicability, such imaging cannot fully capture the three-dimensional spatial relationship between the tibial component, anterior cortex, and intramedullary canal. Future studies incorporating multi-institutional cohorts, three-dimensional CT-based modeling, and clinically meaningful thresholds for nail feasibility are warranted to further refine preoperative assessment. Prospective investigations correlating radiographic parameters with intraoperative findings and postoperative clinical outcomes would be necessary to determine whether these angular measurements can reliably guide surgical decision-making.

In conclusion, intramedullary nailing for tibial shaft fractures distal to TKA may be radiographically feasible in selected cases, with a mean nail corridor that may accommodate currently available tibial nail designs in selected cases. The superior and inferior tibial tuberosity angles were identified as anatomical parameters associated with the radiographic nail corridor. When interpreted within the inherent limitations of a two-dimensional radiographic analysis, these measurements may serve as useful adjuncts in preoperative planning rather than definitive determinants of IMN feasibility.

## Figures and Tables

**Table 1:** Demographics, implant characteristics, and radiographic parameters of the study population.

Variable	Overall (n = 248)	Persona (n = 228)	GKS (n = 20)
Age (years)	74.11 ± 6.97		
Sex			
Male	60 (24.2%)		
Female	188 (75.8%)		
BMI	22.00 ± 4.33		
Implant			
PERSONA	228 (91.9%)		
GKS	20 (8.1%)		
Tibia size			
B		6 (2.6%)	5 (25%)
C		52 (22.8%)	8 (40%)
D		89 (39%)	3 (15%)
E		35 (15.4%)	4 (20%)
F		31 (13.6%)	
G		14 (6.1%)	
H		1 (0.4%)	
Keel length			
28 mm		6 (2.6%)	
40 mm		141 (61.8%)	
50 mm		81 (35.5%)	
32mm			13 (65.0%)
33.5mm			7 (35.0%)
Superior tibial tuberosity angle (°)	105.88 ± 5.91		
Inferior tibial tuberosity angle (°)	155.79 ± 5.71		
Nail corridor (mm)	9.27 ± 2.41		

## Data Availability

The data supporting the findings of this study are not publicly available due to privacy and ethical restrictions but are available from the corresponding author upon reasonable request.

## Supplementary Materials

The following supporting information can be downloaded at: https://www.mdpi.com/article/10.3390/jcm15051801/s1, Figure S1: Superior Tibial Tuberosity Angle vs. Corridor; Figure S2: Corridor vs. Inferior Tibial Tuberosity Angle; Figure S3: Normal Q–Q Plot of residuals for the final regression model.
