# Supplementary figures and images for: Radiographic Verification of the Feasibility of Intramedullary Nailing in Tibial Shaft Fractures Distal to Total Knee Arthroplasty
Source: J Clin Med. 2026 Feb 27;15(5):1801. doi: 10.3390/jcm15051801 (PMC12986093; doi:10.3390/jcm15051801)

# Superior Tibial Tuberosity Angle vs. Corridor

...: Corridor =  $-4.26 + 0.13 \times \text{Superior}$  ( $p < 0.001$ )

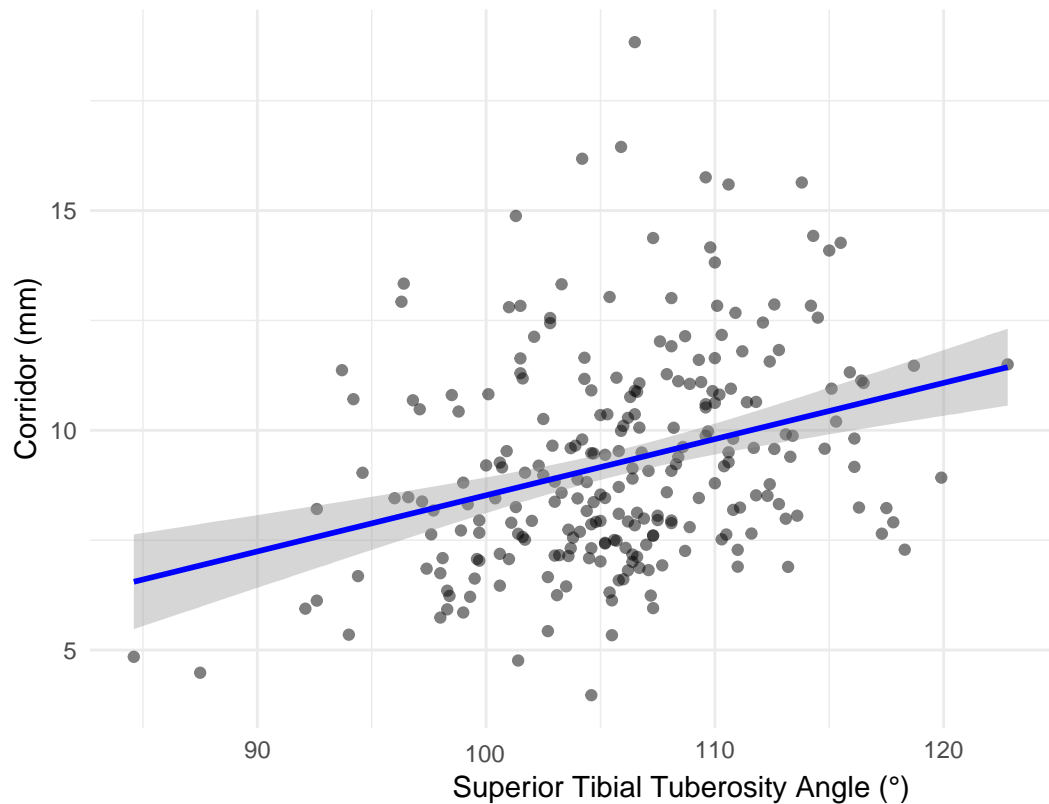

Supplement: Supplementary file 1 [file jcm-15-01801-s001.zip › Figure S1_Scatterplot showing the relationship between superior angle and nail corridor.pdf]

# Corridor vs. Inferior Tibial Tuberosity Angle

Corridor =  $21.18 - 0.076 \times \text{Inferior}$  ( $p = 0.004$ )

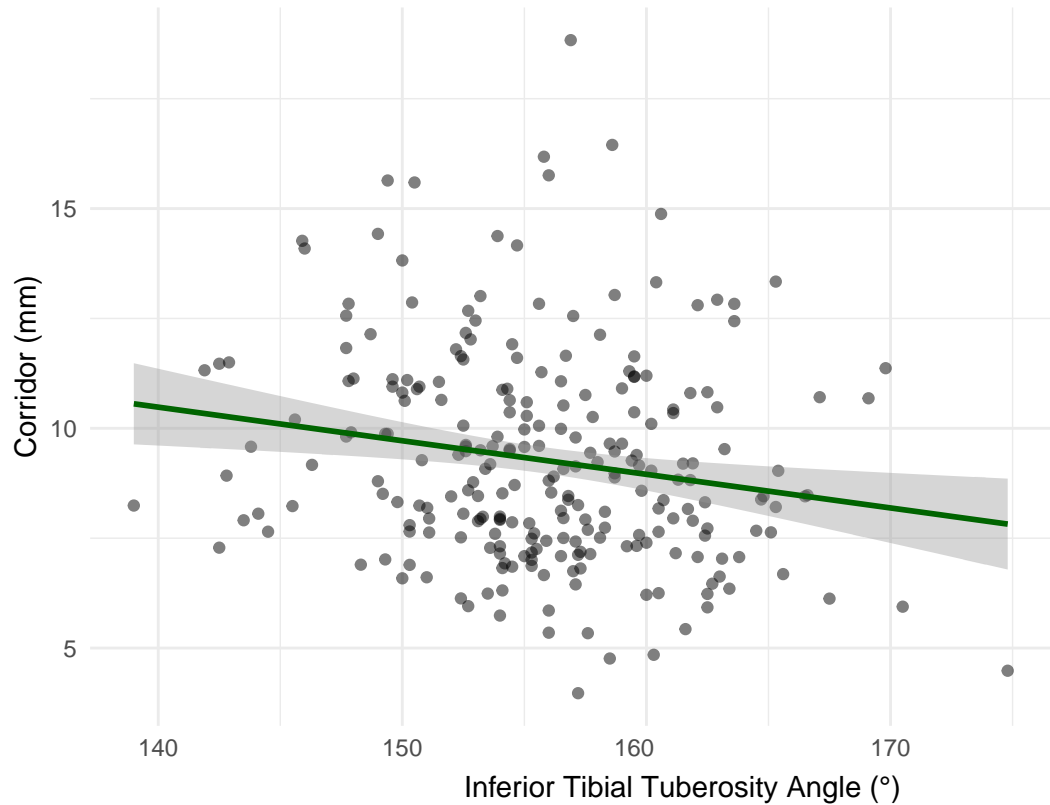

Supplement: Supplementary file 1 [file jcm-15-01801-s001.zip › Figure S2_Scatterplot showing the relationship between inferior angle and nail corridor.pdf]

**Normal Q-Q Plot**

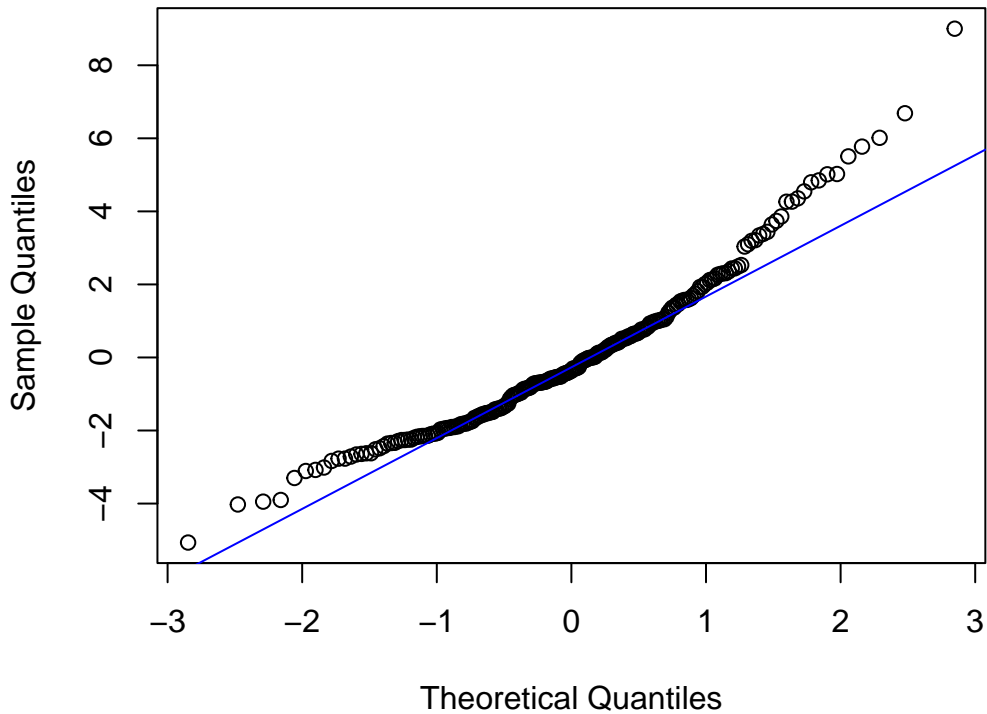

Supplement: Supplementary file 1 [file jcm-15-01801-s001.zip › Figure S3_Q–Q plot of residuals for the final regression model.pdf]
